# Distinguishing the offensive and defensive performances of starters and substitutes between high-level and low-level teams in elite women’s basketball games

**DOI:** 10.1371/journal.pone.0335318

**Published:** 2025-10-24

**Authors:** Wenping Sun, LianYee Kok, ChenSoon Chee, Wensheng Xiao

**Affiliations:** 1 Department of Sports, Nanyang Institute of Technology, Nanyang, China; 2 Department of Sport Science, Faculty of Applied Sciences, Tunku Abdul Rahman University of Management and Technology, Kuala Lumpur, Malaysia; 3 Department of Sport Studies, Faculty of Educational Studies, Universiti Putra Malaysia, Kuala Lumpur, Malaysia; 4 School of Physical Education, Huzhou University, Huzhou, China; 5 College of Physical Education, Hunan Normal University, Changsha, China; Universidade Federal de Goias, BRAZIL

## Abstract

The primary goals of this study were to differentiate the offensive and defensive performances of starters and substitutes between high- and low-level teams in elite women’s basketball. This study examined 432 players from 36 women’s basketball teams who competed in the Tokyo Olympics, the 2022 Women’s Basketball World Cup, and the Paris Olympics. The rank-sum ratio was used to quantify and describe offensive and defensive performances of starters and substitutes, while the independent sample *t*-test, Pearson correlation, and Spearman Rho correlation were employed to test the differences and relationships between the various variables at a 0.05 level of significance. The results indicated that the offensive and defensive performances of both starters (**r* *= 0.932) and substitutes (**r* *= 0.848) had a significant correlation with the final rankings. Compared to high-level teams, low-level teams showed significant differences in points (p* *= 0.017), two-point (p* *= 0.015) and three-point (p* *= 0.000) shooting percentages, assists (p* *= 0.004), and steals (p* *= 0.040) for starters, as well as points (p* *= 0.033) and defensive rebounding (p* *= 0.027) for substitutes. Additionally, both starters and substitutes significantly correlated with points, two-point and three-point shooting percentages, defensive rebounds, and assists (**r* *> 0.600).

## Introduction

In basketball, an intermittent, high-intensity team sport, team performance is the result of the interaction of technical, tactical, physical, psychological, and physiological factors [1,2]. Because of this, the important task of basketball performance analysis is to try to explain and quantify these influences in order to improve performance [1]. In recent years, with the increasing interest in women’s basketball, many researchers have analyzed the performance of women’s basketball players as well as teams from different aspects [3], including but not limited to anthropometric [4], fitness [5], psychological [6], physiological [7–9], and training methods [10,11].

In addition, basketball is a sport that emphasizes strategy, with coaches implementing different offensive and defensive tactics based on the opponents in the game [2,12]. In view of this, previous studies have confirmed the importance of inside-out tactics in elite women’s basketball games [13], as well as fast-break tactics [14]. Furthermore, most studies on technical performance have focused on game-related statistics from women’s basketball [15], as these performance indicators, such as rebounds, assists, and steals, may be utilized to assess and quantify the technical performance of players and teams [1,16]. Consequently, numerous studies have found different performance indicators that distinguish winning teams from losing teams at various women’s basketball levels and ages [17,18]. Furthermore, in women’s basketball games, it has also been verified that when defensive pressure is applied, the opponent’s passing and dribbling execution becomes less efficient [19], and their shooting percentage also drops [20]. However, compared to men’s basketball, women’s basketball’s technical performance components have received less research [18].

In fact, the focus of basketball performance analysis has actually changed over time, with many researchers shifting their attention from team performance to specific roles like starters, substitutes, and different playing positions [21]. In basketball, players’ roles are defined as starters and substitutes based on the order in which they play [22]. It has been shown that the increasing intensity of high-level basketball games like the EuroBasket and the NBA in recent years has led to an increase in player rotation [23], and the performance of substitutes has been cited as a possible factor in a team’s victory in elite basketball [13]. Therefore, many scholars have also analyzed the differences between starters and substitutes in women’s basketball from different perspectives, including game demands [22], aerobic capacity [7], physical performance [23], and performance indicators [13].

The two teams in the basketball game constantly switch between the two fundamental forms of basketball, which are offense and defense [8,24]. Thus, a team’s or player’s strength in the game is reflected in their offensive and defensive performances [25]. A large number of performance indicators, such as points, rebounds, assists, steals, etc., in basketball constitute the offensive and defensive performance of a team or player, which can be reflected by quantifying these game-related data [10]. Although prior evaluations of game-related data or performance indicators can help teams perform better in basketball, they do not accurately represent a team’s or player’s overall strength in the game. Additionally, to the best of our knowledge, previous studies have only explored variations in specific performance indicators between starters and substitutes on women’s basketball teams [13]. However, little is known regarding the disparities in offensive and defensive performance of starters between high- and low-level teams in elite women’s basketball, as well as substitutes.

Therefore, this study aims to examine the differences in offensive and defensive performance between high-level and low-level teams in elite women’s basketball games, focusing on both starters and substitutes. Additionally, this study seeks to determine the relationship between the offensive and defensive performance of starters and substitutes and the final competition rankings, as well as their correlation with various performance indicators. This study hypothesizes that the offensive and defensive performance of both starters and substitutes has a significant relationship with the final competition rankings and each performance indicator, and there is a significant difference in the offensive and defensive performances for starters between high- and low-level teams, as well as for substitutes.

## Materials and methods

### Sample

The last three international elite women’s basketball tournaments were the 2021 Tokyo Olympics, the 2022 Women’s Basketball World Cup, and the 2024 Paris Olympics. The final rankings for each team in these three competitions were shown in Table 1. There was a total of 432 players from 36 teams in the three competitions, and they were divided into two groups according to their roles on the field, i.e., starters and substitutes, as the sample. The starters were the five players who started the game on the court, and the rest of the players were substitutes [22]. The role of each player (starter or substitute) was identified from the official website of the International Basketball Federation [26], where each player was marked as a starter or substitute in the box-score after each game.

**Table 1:** The final rankings for each team in the last three international elite women’s basketball competitions.

Final rankings	Tokyo Olympics	2022 Women’s Basketball World Cup	Paris Olympics
1	USA	USA	USA
2	Japan	China	France
3	France	Australia	Australia
4	Serbia	Canada	Belgium
5	China	Belgium	Spain
6	Spain	Serbia	Serbia
7	Belgium	France	Germany
8	Australia	Puerto Rico	Nigeria
9	Canada	Japan	China
10	Korea	Korea	Puerto Rico
11	Nigeria	Mali	Canada
12	Puerto Rico	Bosnia and Herzegovina	Japan

Note. The information was from the official website of the Fédération International de Basketball Amateur (FIBA) (https://www.fiba.basketball/).

This study was conducted based on the game-related statistics of starters and substitutes, involving a total of 90 matches in the three elite women’s basketball tournaments. However, previous studies suggested that a player who averages 10 minutes of play was considered a team’s primary rotation player [21,27]. As a result, the game-related data of players who less than 10 minutes of playing time was excluded from the sample in this study. The final game-related data contained 889 records for starters and 667 records for substitutes.

### Data collection

We collected game-related data contained 889 records for starters and 667 records for substitutes from three international elite women’s tournaments (Tokyo Olympics, 2022 Women’s Basketball World Cup, and Paris Olympics) on the official website of the Fédération International de Basketball Amateur (https://www.fiba.basketball/) [26]. The game-related data includes offensive performance indicators such as points (PTS), offensive rebounds (OR), 2-point field goal made percentage (2P%), 3-point field goal made percentage (3P%), free-throw field goal made percentage (FT%), turnovers (To), and assists (As), as well as defensive performance indicators such as defensive rebounds (DR), steals (St), blocks (Bl), and personal fouls (PF) [28,29].

The procedure for data collection in this study was as follows: (i) identification of starters and substitutes based on the information provided on the box-score page for each game on the FIBA website, (ii) extraction of game-related scores of each player for each performance indicator, (iii) categorization of teams with the same rankings in the three competitions according to starters and substitutes, and (iv) normalization of game-related data for starters and substitutes. The reason for normalizing the data is that each player’s playing time is different, resulting in different game-related data. However, previous studies have shown that statistics on a per-minute basis tend to be fairly consistent even when players’ playing time is variable [30]. Therefore, players’ game-related data was normalized in this study on a per-40-minute basis because basketball games in both the Olympics and the Women’s Basketball World Cup last 40 minutes [30]. The normalized formula for player data is:


$$\text{OS}\text{4}{\text{0}}_{A}\;=\text{O}{\text{S}}_{A}\;/{\text{MIN}}_{A}\;\times \;\text{4}\text{0},$$

where OS40_*A*_ is normalized to reflect per-40-minute statistics for player *A*, OS_*A*_ is original statistics for player *A*, and MIN_*A*_ is minutes on the court for player *A*.

To ensure the accuracy and consistency of the game-related data, a sub-sample of 15 games were randomly selected from a total of 90 games and observed by two analysts with more than five years of experience in analyzing basketball performance. The results were compared with the data collected from the FIBA official website, and it was found that perfect Intra-class Correlation Coefficients (ICC = 1.0) were obtained for points, free-throw field goal made percentage, 2-point field goal made percentage, 3-point field goal made percentage, rebounds, assists, and blocks. As for turnovers, steals, and personal fouls, the results were lower but still very valid (ICC = 0.93).

### Data analysis

In this study, the 36 teams in the three elite women’s basketball tournaments were grouped according to their final rankings, and teams with the same rankings were grouped together into a total of 12 groups. Players were then further categorized into starters and substitutes within each group. The rank-sum ratio (RSR) method was employed to integrate performance indicators to quantify offensive and defensive performance of starters and substitutes. The RSR approach has several uses in basketball, which advances the science and variety of data analysis and gives coaches additional ways to comprehend information about their opponents and their own squad [28,29]. The formula for calculating RSR is:


RSR=∑R/(M*N),

where M is the number of performance indicators, N is the number of teams, R is the rank value of each indicator, and ∑R is the rank sum of all performance indicators. However, for the R value, certain indicators are coded from small to large indices when performance is better when numbers are larger such as for points and rebounds, while indices for turnovers and fouls are inversely assigned, meaning that the higher the value, the worse the rank [28,29]. When some indicators achieve the same ranks, the mean of these index values is determined. RSR values range from 0 to 1, where higher numbers denote superior performance or ranking [28,29]. Using the 5-level RSR evaluation criteria, this study classified offensive-defensive performances into five categories (A, B, C, D, E) based on RSR values: very strong (≥ 0.8), strong (0.60–0.79), moderate (0.40–0.59), weak (0.20–0.39), and very weak (≤ 0.19).

In addition, this study applied independent sample *t*-test (IBM SPSS Statistics Version 25) to examine the differences in offensive and defensive performance of starters and substitutes between high-level teams (top four groups) and low-level teams (bottom four groups), thereby exploring the characteristics of offensive and defensive performance for high-level teams. Besides that, Spearman rank correlation and Pearson correlation were conducted at the 0.05 significance level to determine the relationship between the offensive and defensive performances of starters and substitutes and the final competition rankings as well as performance indicators. All statistical tests utilizing SPSS software were run bootstrapping with 95% confidence intervals.

## Results

### Analysis of offensive and defensive performances for starters and substitutes in three elite women’s basketball tournaments

The RSR method was employed to evaluate the offensive and defensive performances of starters and substitutes in three elite women’s basketball tournaments. As shown in Tables 2 and 3, the offensive and defensive RSR values, grades, and ranks of starters and substitutes of the different ranked teams in the three elite women’s tournaments were obtained by running the RSR formula.

**Table 2:** The offensive and defensive performances by starters in three elite women’s basketball tournaments.

FR	PTS	R	2P %	R	3P %	R	FT %	R	OR	R	DR	R	As	R	PF	R	To	R	St	R	Bl	R	RSR	Grade	Rank
1	19.27	12	58.31	12	34.52	10	78.20	6	2.11	10	7.23	12	5.47	12	2.02	12	2.49	10	2.08	12	1.49	12	0.91	A	1
2	16.40	11	53.49	11	37.16	12	79.09	8	1.55	4	4.91	4	4.93	11	2.61	10	2.38	12	1.77	8	0.38	2	0.70	B	3
3	15.30	8	48.42	8	34.35	9	81.72	11	1.75	7	5.64	11	4.71	10	3.02	6	2.87	6	1.78	9	0.78	9	0.71	B	2
4	15.28	7	47.84	7	35.14	11	77.40	5	1.62	6	5.32	8	3.97	6	3.29	3	2.91	5	1.46	4	0.80	10	0.55	C	7
5	15.34	9	50.13	9	32.73	7	80.67	10	1.99	9	5.35	9	4.69	9	2.46	11	3.12	3	1.61	5	0.57	6	0.66	B	4
6	14.78	5	42.89	4	33.14	8	78.38	7	2.16	11	5.15	7	4.09	7	3.03	5	2.77	7	2.04	11	0.56	5	0.58	C	6
7	15.56	10	50.54	10	30.92	6	83.45	12	1.15	1	5.36	10	4.17	8	2.68	9	3.06	4	1.99	10	0.58	7	0.66	B	4
8	14.79	6	43.95	5	30.88	5	76.06	4	1.50	3	4.89	3	3.52	4	3.19	4	2.74	8	1.65	6.5	0.40	3	0.39	D	9
9	13.74	3	46.69	6	30.34	4	73.48	2	1.87	8	5.07	6	3.44	3	2.69	8	2.66	9	1.18	2	0.48	4	0.42	C	8
10	13.88	4	40.82	2	28.83	3	75.00	3	1.61	5	4.66	2	3.53	5	3.30	2	2.41	11	1.32	3	0.63	8	0.36	D	10
11	12.90	1	38.99	1	28.17	2	67.54	1	2.32	12	5.04	5	3.16	2	3.33	1	3.62	1	1.65	6.5	0.81	11	0.33	D	11
12	13.02	2	41.64	3	27.01	1	79.27	9	1.25	2	4.23	1	3.15	1	2.77	7	3.23	2	1.08	1	0.27	1	0.23	D	12

Note. All statistics are per 40 minutes at the center position. FR (The final rankings during three elite women’s basketball competitions), PTS (Points), 2P% (2-point field goal made percentage), 3P% (3-point field goal made percentage), FT% (Free throws field goal made percentage), OR (Offensive Rebounds), As (Assists), To (Turnovers), DR (Defensive Rebounds), PF (Personal Fouls), St (Steals), Bl (Blocks).

**Table 3:** The offensive and defensive performances by substitutes in three elite women’s basketball tournaments.

FR	PTS	R	2P %	R	3P %	R	FT %	R	OR	R	DR	R	As	R	PF	R	To	R	St	R	Bl	R	RSR	Grade	Rank
1	16.84	12	51.86	11	40.00	12	83.22	11	1.99	9	5.22	12	5.32	12	3.06	10	3.25	1	1.59	7.5	0.60	4	0.77	B	1
2	15.39	10	48.66	9	33.76	8	79.61	9	1.89	6	4.37	5	3.70	8	3.24	8	2.16	10.5	1.58	6	0.70	9	0.67	B	3
3	16.58	11	54.12	12	37.64	11	80.61	10	1.70	4	4.86	9	4.15	11	3.46	7	2.33	7	1.19	1	0.84	12	0.72	B	2
4	13.46	5	41.16	3	24.47	1	65.38	1	2.12	10	4.17	4	2.85	4	3.18	9	2.16	10.5	1.79	9	0.77	10	0.50	C	7
5	14.38	9	48.82	10	36.94	10	76.83	6	1.31	1	5.19	11	4.13	10	3.76	6	3.15	2	1.96	11	0.69	8	0.64	B	4
6	13.39	4	48.00	8	29.49	4	67.16	2	1.93	8	4.51	7	3.46	7	4.25	1	2.63	6	1.36	2	0.66	6	0.42	C	10
7	12.66	3	47.15	6	30.30	5	85.71	12	1.90	7	4.92	10	2.27	2	3.93	4	1.97	12	2.21	12	0.80	11	0.64	B	4
8	13.65	7	41.25	4	33.78	9	76.92	7	2.39	11	4.62	8	2.76	3	2.97	11	3.03	3	1.59	7.5	0.48	3	0.56	C	6
9	14.18	8	44.74	5	28.69	3	77.78	8	1.86	5	4.40	6	3.81	9	3.91	5	2.20	9	1.42	4	0.44	2	0.48	C	8
10	11.76	2	47.58	7	32.79	7	72.73	4	1.64	3	3.09	2	2.91	5.5	4.18	2	2.30	8	1.39	3	0.67	7	0.38	D	11
11	13.62	6	37.34	2	25.00	2	75.00	5	2.72	12	3.48	3	2.26	1	2.72	12	2.96	4	1.45	5	0.64	5	0.43	C	9
12	11.71	1	36.46	1	32.00	6	67.86	3	1.45	2	2.91	1	2.91	5.5	4.00	3	2.84	5	1.89	10	0.36	1	0.29	D	12

Note. All statistics are per 40 minutes at the center position. FR (The final rankings during three elite women’s basketball competitions), PTS (Points), 2P% (2-point field goal made percentage), 3P% (3-point field goal made percentage), FT% (Free throws field goal made percentage), OR (Offensive Rebounds), As (Assists), To (Turnovers), DR (Defensive Rebounds), PF (Personal Fouls), St (Steals), Bl (Blocks).

### Relationship between the offensive and defensive performances of starters and substitutes and the final rankings

The Spearman Rho correlation was used to test the correlation between the offensive and defensive performances of starters and substitutes and the final rankings, respectively. The final competition rankings during the three elite women’s basketball tournaments were the dependent variables, and the offensive and defensive RSR rankings of starters and substitutes among the 12 groups of teams were the independent variables. The results indicated that there was a significant and positive correlation between offensive and defensive performance at both starters (p* *= 0.000, **r* *= 0.932) and substitutes (p* *= 0.000, **r* *= 0.848) and the final rankings.

### Analysis of the differences in offensive and defensive performances of starters and substitutes between the high-level and low-level teams

The top four teams in the three elite women’s basketball competitions represent the high-level teams, while the bottom four teams represent the low-level teams. The top four and bottom four teams were used as independent variables, and the offensive and defensive performances of starters and substitutes were dependent variables, respectively. The independent sample *t*-test was conducted to examine the difference between variables, and the results showed a very significant difference between the offensive and defensive performances of high-level and low-level teams, both in terms of starters (p* *= 0.004) and substitutes (p* *= 0.009) (Table 4).

**Table 4:** Differences in offensive and defensive performances between the top four and bottom four teams for starters and substitutes in the three elite women’s basketball tournaments.

	The RSR values of starters	The RSR values of substitutes
Top four (x ± S_d_)	0.72 ± 0.15	0.67 ± 0.12
Bottom four (x ± S_d_)	0.34 ± 0.08	0.40 ± 0.08
Difference	0.38	0.27
T	4.562	3.787
P (2-tailed)	0.004**	0.009**

Note. ** p < 0.01, with a very significant difference.

The variability of each offensive and defensive indicator was analyzed in order to better investigate the precise differences between high-level and low-level teams with regard to starters and substitutes (Table 5). The top four and bottom four teams were independent variables, and the offensive and defensive indicators of starters and substitutes were dependent variables, respectively. The results indicated that there was a significant difference in PTS (p* *= 0.017), 2P% (p* *= 0.015), 3P% (p* *= 0.000), assists (p* *= 0.004), and steals (p* *= 0.040) of starters between high-level and low-level teams, as well as for substitutes’ PTS (p* *= 0.033) and defensive rebounds (p* *= 0.027).

**Table 5:** Differences in offensive and defensive indicators between the top four and bottom four teams for starters and substitutes in the three elite women’s basketball tournaments.

The starters											
	PTS	2P %	3P %	FT %	OR	As	To	DR	St	Bl	PF
Top four (x ± S_d_)	16.6 ± 1.9	52.0 ± 4.9	35.3 ± 1.3	79.1 ± 1.9	1.8 ± 0.2	4.8 ± 0.6	2.7 ± 0.3	5.8 ± 1.0	1.8 ± 0.3	0.9 ± 0.5	2.7 ± 0.6
Bottom four (x ± S_d_)	13.4 ± 0.5	42.0 ± 3.3	28.6 ± 1.4	73.8 ± 4.9	1.8 ± 0.5	3.3 ± 0.2	3.0 ± 0.5	4.8 ± 0.4	1.3 ± 0.2	0.5 ± 0.2	3.0 ± 0.3
Difference	3.2	10.0	6.7	5.3	0.0	1.5	−0.3	1.0	0.5	0.4	−0.3
T	3.269	3.378	7.071	2.030	−0.019	4.453	−1.042	1.883	2.621	1.224	−0.887
P (2-tailed)	0.017^*^	0.015^*^	0.000^**^	0.089	0.985	0.004^**^	0.337	0.109	0.040^*^	0267	0.409
**The substitutes**											
	PTS	2P %	3P %	FT %	OR	As	To	DR	St	Bl	PF
Top four (x ± S_d_)	15.6 ± 1.5	49.0 ± 5.7	34.0 ± 6.8	77.2 ± 8.0	1.9 ± 0.2	4.0 ± 1.0	2.5 ± 0.5	4.7 ± 0.5	1.5 ± 0.3	0.7 ± 0.1	3.2 ± 0.2
Bottom four (x ± S_d_)	12.8 ± 1.3	41.5 ± 5.5	29.6 ± 3.6	73.3 ± 4.2	1.9 ± 0.6	3.0 ± 0.6	2.6 ± 0.4	3.5 ± 0.7	1.5 ± 0.2	0.5 ± 0.2	3.7 ± 0.7
Difference	2.8	7.5	4.4	3.9	0.0	1.0	−0.1	1.2	0.0	0.2	−0.5
T	2.754	1.884	1.129	0.853	0.026	1.706	−0.309	2.902	0.000	2.189	−1.364
P (2-tailed)	0.033^*^	0.109	0.302	0.427	0.980	0.139	0.768	0.027^*^	1.000	0.071	0.221

Note. * p < 0.05, with a significant difference. ** p < 0.01, with a very significant difference.

### Relationship between the offensive and defensive performances of starters and substitutes and performance indicators

Pearson correlation analysis was employed to investigate the relationship between the variables, with each performance indicator serving as the independent variable and the offensive and defensive performances of starters and substitutes as the dependent variables. The results showed PTS (p* *= 0.000, **r* *= 0.913), 2P% (p* *= 0.000, **r* *= 0.897), 3P% (p* *= 0.001, **r* *= 0.815), DR (p* *= 0.001, **r* *= 0.826), As (p* *= 0.000, **r* *= 0.969), PF (p* *= 0.024, **r* *= 0.644), St (p* *= 0.004, **r* *= 0.763), and Bl (p* *= 0.038, **r* *= 0.603) had a significant correlation with the offensive and defensive performances of starters (Figure in S1 Fig). For the substitutes, the offensive and defensive performances indicated significant correlations with PTS (p* *= 0.001, **r* *= 0.838), 2P% (p* *= 0.007, **r* *= 0.730), 3P% (p* *= 0.030, **r* *= 0.624), FT% (p* *= 0.003, **r* *= 0.771), DR (p* *= 0.000, **r* *= 0.858), and As (p* *= 0.038, **r* *= 0.602) (Figure in S1 Fig).

## Discussion

This study examined the last three international elite women’s basketball tournaments and identified substantial disparities in offensive and defensive performance of both starters and substitutes between high-level and low-level women’s basketball teams. These differences pertain to the points, shooting percentage (both two-pointers and three-point field goals), assists, and steals of starters, as well as the points and defensive rebounding of substitutes. Additionally, the offensive and defensive performance of both starters and substitutes had a significant and positive correlation with the final rankings in all three tournaments. Each performance indicator impacted the offensive and defensive performance of starters and substitutes variably; however, points, two-point field goal percentage, three-point field goal percentage, defensive rebounds, and assists exhibited a significant correlation with the performance of both player categories.

Player rotation during matches is considered a positive strategy to optimize team performance because players are inevitably affected by factors such as injuries, fatigue, or fouls [31]. In many studies on men’s basketball, the performance of substitutes was considered a key factor in distinguishing winning from losing teams [27]. The current study on elite women’s basketball games has indicated that substitutes’ offensive and defensive performance was also an important factor in differentiating high-level teams from low-level ones. The distinction, however, was that starters’ offensive and defensive performance was also a significant determining element. This suggests that there is a considerable overall competitive strength disparity between teams in today’s elite women’s basketball games, as seen by game score differences. According to a previous study, basketball games with a final score differential of more than 12 points between the two teams are unbalanced [17]. In this example, 61 of the 90 games in the three women’s basketball competitions were unbalanced, accounting for around 68%. In comparison, 49% of unbalanced games in men’s competitions occurred during the same time span [26].

Furthermore, the majority of research on game-related data has concentrated on determining the characteristics that distinguish winning teams from losing or successful teams from failing; studies on women’s basketball are no exception [13,18]. These studies certainly provided coaches with important information about the game performance of women’s basketball players and teams [18]. Basketball is a sport in which the ultimate score determines the winner and the loser, and shooting is one of the most important skills in the game of basketball [20]. Unsurprisingly, shooting percentage is a key factor in determining the outcome of a game and an important aspect of basketball performance [18,32]. Specifically, previous studies on the women’s basketball game have consistently confirmed that two-point field goal percentage has a significant positive correlation with game outcomes, whether in intercontinental tournaments or across different levels or ages [15,16,18]. This study further validated this idea by finding that two-point field goal percentage in elite women’s basketball games had a significant correlation with both the offensive and defensive performance of starters and substitutes. Additionally, this study further revealed that the poor two-point shooting accuracy of starting players in low-level teams was one of the main reasons for the team’s failure to achieve better results.

Free throws were considered one of the most efficient ways to score because they were shots taken without defense and were more controlled and consistent [1]. The Spanish Women’s Professional League [17], the Chinese Women’s Basketball League [1], and the Olympic Women’s Basketball Games [16] all showed that free throw shooting percentage had a significant influence on game results. However, the findings of this study did not support this view. Research indicated that female basketball players’ performance in free throws can be influenced by anxiety and attention levels [33], and this psychological uncertainty may be a reason for the inconsistent research results. The three-point field goal percentage was reported to not be significantly related to game outcomes in the Chinese Women’s Basketball League [1]. However, different conclusions were found in other elite women’s basketball tournaments [18]. This study agreed and further argued that the success of the women’s basketball team was largely attributed to the three-point field goal shooting percentage of the starters. Prior studies have demonstrated that when defensive pressure is high, shot percentage dramatically drops [20]. Consequently, the low shooting percentages of starters on the low-level teams in this study may have been caused by the considerable disparity in the defensive statistic of steals between their starters and those on the high-level teams.

In addition, previous studies have also revealed the importance of defensive rebounds for the success of both women’s and men’s basketball teams [16,21]. Defensive rebounds are the main source of fast-break scores and a vital component in a team’s transition from defense to offense [18,21]. The present study agreed with this view and concluded that the main difference in defensive rebounding between high-level and low-level teams was reflected only in the performance of the substitutes. The possible reason is that in many unbalanced games, high-level teams may benefit from a strategic rotation that allows substitutes to gain more valuable experience and contribute to critical game statistics. Additionally, offensive rebounds have also been confirmed in some studies of women’s basketball games as an important indicator for distinguishing between winning and losing [1,18]. The reason is that winning women’s basketball teams exhibit higher second-chance points, and offensive rebounds provide opportunities to create second-chance points [34]. However, this study did not find this characteristic among starters and substitutes.

In basketball, assists were also seen as an important factor in victory in women’s basketball [16], as well as one of the most important indicators to differentiate between starters and substitutes [35]. Studies have indicated that high-level teams win games primarily due to the assists of their starters [36], and that assists can distinguish between the top and bottom teams in women’s basketball1, which is similar to the findings of this study. The explanation for this result is that starters tend to be better at making quick and correct decisions to pass the ball to an unguarded teammate in time to score [36]. In addition, steals are a major indicator of defensive performance in basketball, and the more steals a team makes, the greater the chances of winning the game [13]. Therefore, steals have been shown to be an important indicator in determining the final outcome of women’s basketball teams [18]. This result was also validated in the present study and was specific to steals by the starters rather than the substitutes. However, a study on the Chinese Women’s Basketball League found no correlation between steals and game outcomes [1]. There was evidence suggesting that the performance of excellent female basketball players in steals and assists was significantly related to certain physical qualities such as speed, agility, and particularly high aerobic capacity [37]. Therefore, it is recommended that lower-level women’s basketball teams incorporate these relevant physical qualities into their players’ daily training.

In fact, current research findings on the impact of various performance indicators on women’s basketball games tend to be inconsistent. The reasons may be differences in sample size, research methods, and competition levels [1], or it could be attributed to female basketball players being more easily influenced by situational variables such as the location of the game and the quality of the opponents [38]. Therefore, the limitations of this study are considered as follows. First, some off-court factors that may impact female basketball players’ performance were not considered in this study, such as psychological pressure and player physical attributes [37]. Second, as a situational variable, opponents’ strength variances during basketball games may also cause players’ game-related data to vary [39]. Future research can categorize and analyze games based on the final score difference to obtain more valuable information about starters and substitutes.

## Conclusion

According to this study, starters and substitutes on today’s women’s national basketball teams performed very differently offensively and defensively, and these variations had a significant impact on the final rankings. In terms of specific performance indicators, points, two-point field goal percentage, three-point field goal percentage, defensive rebounds, and assists correlated significantly with the offensive and defensive performances of both starters and substitutes and were also found to be the primary factors influencing the outcome of elite women’s basketball games. For low-level women’s basketball teams, it is necessary to strengthen the shooting practice of starters under high-intensity defensive conditions. Additionally, certain physical qualities of starters, such as speed, agility, and aerobic capacity, should be enhanced to improve their assist and steal abilities, or players with these characteristics should be adjusted into the starting lineup. Furthermore, substitutes need to improve their defensive capabilities, primarily in defending rebounds.

## Supporting information

S1 Fig Correlation between the offensive-defensive performances of starters and substitutes and performance indicators. (PDF)
