# Supplementary material for: Distinguishing the offensive and defensive performances of starters and substitutes between high-level and low-level teams in elite women’s basketball games
Source: PLoS One. 2025 Oct 24;20(10):e0335318. doi: 10.1371/journal.pone.0335318 (PMC12551822; doi:10.1371/journal.pone.0335318)
Supplement: S1 Fig — (PDF) [file pone.0335318.s001.pdf]

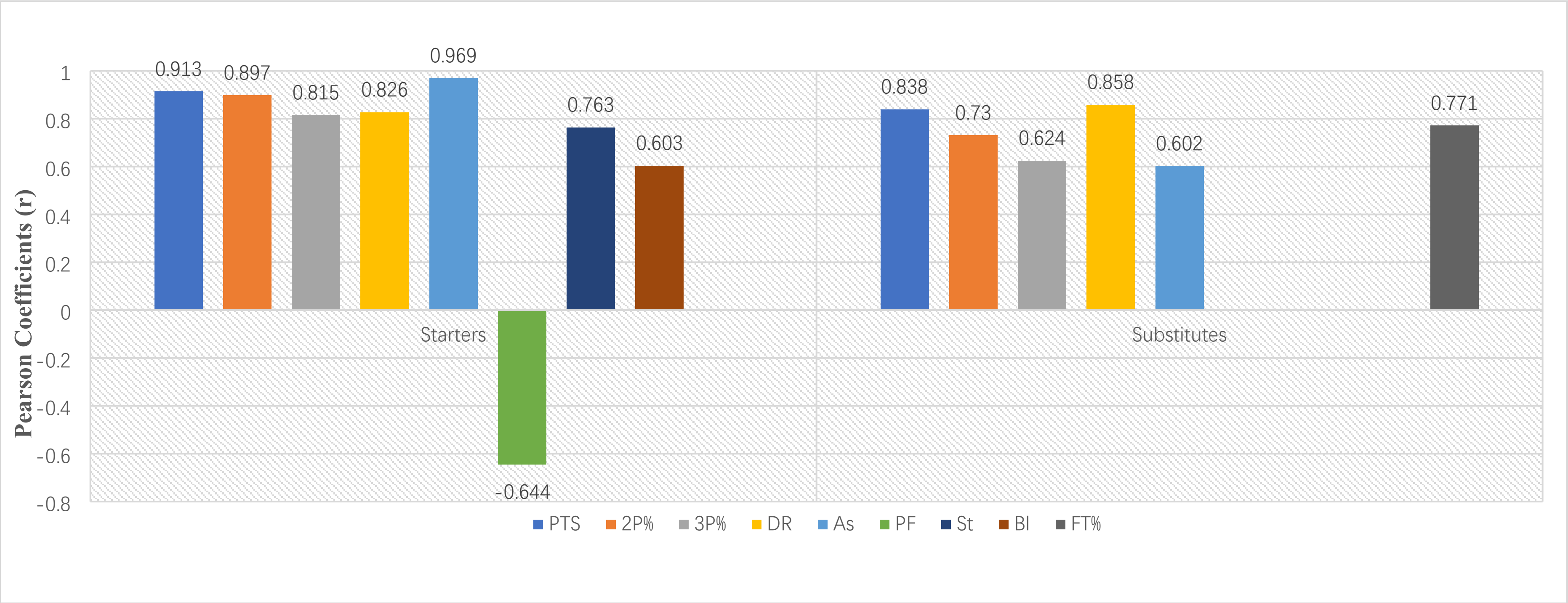

Figure 1. Correlation between the offensive-defensive performances of starters and substitutes and performance indicators
